# Supplementary material for: The Role of Molossidae and Vespertilionidae in Shaping the Diversity of Alphacoronaviruses in the Americas
Source: Microbiol Spectr. 2022 Oct 12;10(6):e03143-22. doi: 10.1128/spectrum.03143-22 (PMC9769993; doi:10.1128/spectrum.03143-22)
Supplement: Supplemental file 1 — Tables S1 to S3. Download spectrum.03143-22-s0001.pdf, PDF file, 0.9 MB [file spectrum.03143-22-s0001.pdf]

Sample ID	Host	Clade	Country	Province	Locality	Latitude	Longitude	Collection Date	ACCN
A488 20	*Molossus molossus*	A	Argentina	Buenos Aires	Berazategui	-34.77	-58.21	2020	OP169153
P176 20	*Tadarida brasiliensis*	A	Argentina	CABA	CABA	-34.60	-58.45	2020	OP169160
P197 20	*Tadarida brasiliensis*	A	Argentina	CABA	CABA	-34.60	-58.45	2020	OP169162
P364 21	*Tadarida brasiliensis*	A	Argentina	Chubut	Trelew	-43.25	-65.31	2021	OP169165
P534 20	*Molossus molossus*	B	Argentina	Buenos Aires	Moreno	-34.63	-58.79	2020	OP169167
P164 20	*Molossus* spp.	B	Argentina	CABA	CABA	-34.60	-58.45	2020	OP169159
P205 20	*Molossus* spp.	B	Argentina	CABA	CABA	-34.60	-58.45	2020	OP169163
A479 20	*Myotis* spp.	B	Argentina	Buenos Aires	San Isidro	-34.47	-58.53	2020	OP169152
P207 20	*Myotis* spp.	B	Argentina	Neuquén	Neuquén	-38.95	-68.06	2020	OP169164
A379 20	*Tadarida brasiliensis*	B	Argentina	Buenos Aires	Balcarce	-37.85	-58.26	2020	OP169150
A415 20	*Tadarida brasiliensis*	B	Argentina	Buenos Aires	Merlo	-34.67	-58.73	2020	OP169151
A533 20	*Tadarida brasiliensis*	B	Argentina	Buenos Aires	Moron	34.58	-58.62	2020	OP169154
A637 20	*Tadarida brasiliensis*	B	Argentina	Buenos Aires	San Martin	-34.43	-58.54	2020	OP169155
P196 20	*Tadarida brasiliensis*	B	Argentina	CABA	CABA	-34.60	-58.45	2020	OP169161
P507 20	*Tadarida brasiliensis*	B	Argentina	CABA	CABA	-34.60	-58.45	2020	OP169166
P111 21	*Tadarida brasiliensis*	B	Argentina	CABA	CABA	-34.60	-58.45	2021	OP169156
P113 21	*Tadarida brasiliensis*	B	Argentina	CABA	CABA	-34.60	-58.45	2021	OP169157
P552 20	*Tadarida brasiliensis*	B	Argentina	La Pampa	Santa Rosa	-36.62	-64.28	2020	OP169168
P125 20	*Tadarida brasiliensis*	B	Argentina	ND	ND	ND	ND	2020	OP169158
P919 20	*Tadarida brasiliensis*	B	Argentina	Neuquén	Neuquén	-38.95	-68.06	2020	OP169169
P934 20	*Tadarida brasiliensis*	B	Argentina	Río Negro	Las Grutas	-40.73	-64.93	2020	OP169170

Supplementary Table S1. Sample ID, host, clade, country, state, locality, latitude, longitude, and Genbank accession numbers of the alphaCoV sequences produced in this study. CABA: Ciudad Autónoma de Buenos Aires

Supplementary Table S2. Sample ID, host, taxonomy, country, and Genbank accession numbers of the CoV sequences retrieved from Genbank and used in this study.

ACCN	Taxonomy	Host	Country	Collection date
NC_022103	Alphacoronavirus	Myotis lucifugus	USA	2006
NC_048216	Alphacoronavirus	Triaenops afer	Kenya	2010
NC_046964	Alphacoronavirus	Pipistrellus kuhlii	Italy	2015
NC_032107	Alphacoronavirus	Triaenops afer	Kenya	2010
NC_028833	Alphacoronavirus	Nyctalus velutinus	China	2013
NC_028824	Alphacoronavirus	Rhinolophus ferrumequinum	China	2012
NC_028814	Alphacoronavirus	Rhinolophus ferrumequinum	China	2013
NC_028811	Alphacoronavirus	Myotis ricketti	China	2011
NC_032730	Alphacoronavirus	Rattus norvegicus	China	2013
NC_018871	Alphacoronavirus	Rousettus leschenaulti	China	2005
NC_025217	Betacoronavirus	Hipposideros pratti	China	2013
NC_030886	Betacoronavirus	Rousettus leschenaulti	China	2014
MN996532	Betacoronavirus	Rhinolophus affinis	China	2013
NC_034440	unclassified	Pipistrellus hesperidus	Uganda	2013
NC_014470	unclassified	Rhinolophus blasii	Bulgaria	2008
NC_010438	unclassified	Miniopterus	China	-
NC_010437	unclassified	Miniopterus	China	-
NC_009988	unclassified	Rhinolophus	-	-
NC_009021	unclassified	Pipistrellus	China	
NC_009020	unclassified	Pipistrellus	China	
NC_009019	unclassified	Tylonycteris	China	
NC_009657	unclassified	Scotophilus	-	-
NC_005831	Alphacoronavirus	Homo sapiens	-	-
NC_002645	Alphacoronavirus	Homo sapiens	-	-
NC_045512	Betacoronavirus	Homo sapiens	China	2019
NC_006213	Betacoronavirus	Homosapiens	USA	-
NC_038294	Betacoronavirus	Homo sapiens	United Kingdom	2012
NC_019843	Betacoronavirus	Homo sapiens	-	2012
NC_006577	Betacoronavirus	Homo sapiens	-	-
NC_004718	Betacoronavirus	Homo sapiens	Canada	-
NC_034972	Alphacoronavirus	Apodemus chevrieri	China	2011
NC_028752	Alphacoronavirus	camel	Saudi Arabia	2015
MT121216	Betacoronavirus	Manis javanica	China	2019
MT040336	Betacoronavirus	Manis javanica	China	2017
NC_039207	Betacoronavirus	Erinaceus europaeus	Germany	2012
NC_026011	Betacoronavirus	Rattus norvegicus	China	2012
NC_017083	Betacoronavirus	Oryctolagus cuniculus	China	2006
NC_012936	Betacoronavirus	rat	-	-
NC_003045	Betacoronavirus	bovine	-	-
KX432213	Betacoronavirus	dog	China	2014
JX860640	Betacoronavirus	dog	South Korea	2008
NC_010646	Gammacoronavirus	Delphinapterus leucas	-	-
NC_038861	Alphacoronavirus	pig	USA	-
NC_030292	Alphacoronavirus	Mustela putorius	Netherlands	2010
NC_028806	Alphacoronavirus	pig	Italy	2009
NC_023760	Alphacoronavirus	Mustela vison	USA	1998

NC_002306	Alphacoronavirus	feline	USA	-
NC_003436	Alphacoronavirus	pig	-	-
NC_039208	Deltacoronavirus	pig	China	2010
NC_010800	Gammacoronavirus	turkey	Canada	-
NC_048214	Gammacoronavirus	duck	China	2014
NC_048213	Gammacoronavirus	chicken	India	2003
NC_046965	Gammacoronavirus	Branta canadensis	Canada	2017
NC_001451	Gammacoronavirus	bird	-	-
NC_011547	Deltacoronavirus	red-whiskered bulbul	Hong Kong	2007
NC_016992	Deltacoronavirus	sparrow	China	2007
NC_016991	Deltacoronavirus	white-eye	China	2007
NC_016996	Deltacoronavirus	common moorhen	China	2007
NC_016995	Deltacoronavirus	wigeon	China	2008
NC_016994	Deltacoronavirus	night-heron	China	2007
NC_016993	Deltacoronavirus	magpie-robin	China	2007
NC_011550	Deltacoronavirus	white-rumped munia	China	Apr-07
NC_011549	Deltacoronavirus	grey-backed thrush	China	Jan-07
NC_001846	Betacoronavirus	mouse	-	-
NC_048217	Betacoronavirus	mouse	-	-
AC_000192	Betacoronavirus	mouse	-	-
JX537914	Alphacoronavirus	Eptesicus fuscus	USA	2009
JQ731793	Alphacoronavirus	Carollia perspicillata	Costa Rica	2010
JQ731792	Alphacoronavirus	Carollia perspicillata	Costa Rica	2010
JQ731784	Alphacoronavirus	Artibeus jamaicensis	Panama	2010
JQ731788	Alphacoronavirus	Artibeus lituratus	Panama	2011
EF544564	Alphacoronavirus	Myotis occultus	USA	2006
KY820805	Alphacoronavirus	Myotis lucifugus	Canada	2010
KY820799	Alphacoronavirus	Myotis lucifugus	Canada	2010
KY820784	Alphacoronavirus	Myotis lucifugus	Canada	2010
KY799179	Alphacoronavirus	Myotis lucifugus	Canada	2010
JX537913	Alphacoronavirus	Perimyotis subflavus	USA	2010
JQ731789	Alphacoronavirus	Carollia perspicillata	Costa Rica	2010
JQ731776	Alphacoronavirus	Anoura geoffroyi	Costa Rica	2010
JQ731783	Alphacoronavirus	Phyllostomus discolor	Panama	2011
JQ731785	Alphacoronavirus	Artibeus jamaicensis	Panama	2010
KM215146	Alphacoronavirus	Glossophaga soricina	Costa Rica	2014
EF544563	Alphacoronavirus	Myotis occultus	USA	2006
KY820806	Alphacoronavirus	Myotis lucifugus	Canada	2010
KY820792	Alphacoronavirus	Myotis lucifugus	Canada	2010
KY820782	Alphacoronavirus	Myotis lucifugus	Canada	2010
KY820777	Alphacoronavirus	Myotis lucifugus	Canada	2010
KY820775	Alphacoronavirus	Myotis lucifugus	Canada	2010
OL415261	Alphacoronavirus	Eptesicus fuscus	USA	2020
JQ731782	Alphacoronavirus	Phyllostomus discolor	Panama	2011
KC779225	Alphacoronavirus	Carollia perspicillata	Costa Rica	2012
EF544565	Alphacoronavirus	Myotis occultus	USA	2006
KY820803	Alphacoronavirus	Myotis lucifugus	Canada	2010
KY820793	Alphacoronavirus	Myotis lucifugus	Canada	2010
KY820769	Alphacoronavirus	Myotis lucifugus	Canada	2010
JQ731801	Alphacoronavirus	Carollia perspicillata	Costa Rica	2010
JQ731790	Alphacoronavirus	Carollia perspicillata	Costa Rica	2010

HQ336976	Alphacoronavirus	Myotis volans	USA	2007
HQ336975	Alphacoronavirus	Myotis volans	USA	2009
EF544567	Alphacoronavirus	Myotis occultus	USA	2006
KX663833	Alphacoronavirus	Tadarida brasiliensis	USA	2016
KY820802	Alphacoronavirus	Myotis lucifugus	Canada	2010
KY820795	Alphacoronavirus	Myotis lucifugus	Canada	2010
KY820794	Alphacoronavirus	Myotis lucifugus	Canada	2010
KY820791	Alphacoronavirus	Myotis lucifugus	Canada	2010
KY820788	Alphacoronavirus	Myotis lucifugus	Canada	2010
KY820785	Alphacoronavirus	Myotis lucifugus	Canada	2010
KY820779	Alphacoronavirus	Myotis lucifugus	Canada	2010
KY820778	Alphacoronavirus	Myotis lucifugus	Canada	2010
KY820774	Alphacoronavirus	Myotis lucifugus	Canada	2010
KY820773	Alphacoronavirus	Myotis lucifugus	Canada	2010
KY820770	Alphacoronavirus	Myotis lucifugus	Canada	2010
OL415262	Alphacoronavirus	Eptesicus fuscus	USA	2021
JQ731777	Alphacoronavirus	Anoura geoffroyi	Costa Rica	2010
KM215147	Alphacoronavirus	Carollia castanea	Costa Rica	2014
KY820783	Alphacoronavirus	Myotis lucifugus	Canada	2010
KY820780	Alphacoronavirus	Myotis lucifugus	Canada	2010
KY820776	Alphacoronavirus	Myotis lucifugus	Canada	2010
KY820772	Alphacoronavirus	Myotis lucifugus	Canada	2010
KY820800	Alphacoronavirus	Myotis lucifugus	Canada	2010
OL410607	Alphacoronavirus	Eptesicus fuscus	USA	2020
OL410608	Alphacoronavirus	Eptesicus fuscus	USA	2020
JQ731787	Alphacoronavirus	Artibeus lituratus	Panama	2010
EF544566	Alphacoronavirus	Eptesicus fuscus	USA	2006
KY820767	Alphacoronavirus	Myotis lucifugus	Canada	2010
KY820807	Alphacoronavirus	Myotis lucifugus	Canada	2010
KY820801	Alphacoronavirus	Myotis lucifugus	Canada	2010
KY820796	Alphacoronavirus	Myotis lucifugus	Canada	2010
KY820789	Alphacoronavirus	Myotis lucifugus	Canada	2010
KY820768	Alphacoronavirus	Myotis lucifugus	Canada	2010
JQ731775	Alphacoronavirus	Anoura geoffroyi	Costa Rica	2010
JQ731778	Alphacoronavirus	Anoura geoffroyi	Costa Rica	2010
KC779226	Alphacoronavirus	Artibeus jamaicensis	Costa Rica	2012
KF430219	Alphacoronavirus	Myotis lucifugus	USA	2006
KY820804	Alphacoronavirus	Myotis lucifugus	Canada	2010
KY820798	Alphacoronavirus	Myotis lucifugus	Canada	2010
KY820797	Alphacoronavirus	Myotis lucifugus	Canada	2010
KY820787	Alphacoronavirus	Myotis lucifugus	Canada	2010
KY820786	Alphacoronavirus	Myotis lucifugus	Canada	2010
KY820781	Alphacoronavirus	Myotis lucifugus	Canada	2010
OL410609	Alphacoronavirus	Eptesicus fuscus	USA	2020
OL410610	Alphacoronavirus	Eptesicus fuscus	USA	2021
JQ731786	Alphacoronavirus	Artibeus jamaicensis	Panama	2010
JQ731791	Alphacoronavirus	Carollia perspicillata	Costa Rica	2010
KY820790	Alphacoronavirus	Myotis lucifugus	Canada	2010
KY820771	Alphacoronavirus	Myotis lucifugus	Canada	2010
MT734810	Alphacoronavirus	Myotis yumanensis	USA	2020
JQ731795	Alphacoronavirus	Carollia brevicauda	Brazil	2009

JQ731797	Alphacoronavirus	Carollia brevicauda	Brazil	2009
KU552078	Alphacoronavirus	Cynomops planirostris	Brazil	2014
KT717392	Alphacoronavirus	Sturnira lilium	Brazil	2012
KT717389	Alphacoronavirus	Molossus rufus	Brazil	2014
KX094984	Alphacoronavirus	Molossus molossus	Brazil	2009
KX285049	Alphacoronavirus	Sturnira erythromos	Bolivia	2011
MH974764	Alphacoronavirus	Molossus rufus	Brazil	2015
MH974765	Alphacoronavirus	Molossus rufus	Brazil	2015
MH974767	Alphacoronavirus	Molossus rufus	Brazil	2017
MH974771	Alphacoronavirus	Molossus rufus	Brazil	2015
MH974776	Alphacoronavirus	Molossus rufus	Brazil	2017
MT671953	Alphacoronavirus	Tadarida brasiliensis	Brazil	2014
MT671958	Alphacoronavirus	Tadarida brasiliensis	Brazil	2014
MN872697	Alphacoronavirus	Carollia perspicillata	Brazil	2012
MN872700	Alphacoronavirus	Carollia perspicillata	Brazil	2012
KU552077	Alphacoronavirus	Cynomops abrasus	Brazil	2013
KT717393	Alphacoronavirus	Sturnira lilium	Brazil	2012
KX285056	Alphacoronavirus	Carollia perspicillata	Brazil	2009
MH974768	Alphacoronavirus	Molossus molossus	Brazil	2016
MH974773	Alphacoronavirus	Molossus molossus	Brazil	2016
MT671952	Alphacoronavirus	Tadarida brasiliensis	Brazil	2014
MN872696	Alphacoronavirus	Sturnira lilium	Brazil	2012
KT717384	Alphacoronavirus	Carollia perspicillata	Brazil	2011
KX285068	Alphacoronavirus	Mesophylla macconnelli	Brazil	2011
KX285069	Alphacoronavirus	Carollia perspicillata	Brazil	2011
MH974779	Alphacoronavirus	Eptesicus sp.	Brazil	2015
MH974780	Alphacoronavirus	Glossophaga soricina	Brazil	2015
MN872699	Alphacoronavirus	Carollia perspicillata	Brazil	2012
JQ731796	Alphacoronavirus	Carollia perspicillata	Brazil	2009
KU552075	Alphacoronavirus	Cynomops abrasus	Brazil	2013
KX285047	Alphacoronavirus	Carollia perspicillata	Bolivia	2011
KX285050	Alphacoronavirus	Carollia perspicillata	Bolivia	2011
KX285061	Alphacoronavirus	Sturnira lilium	Brazil	2010
KX285062	Alphacoronavirus	Artibeus lituratus	Brazil	2010
MH974775	Alphacoronavirus	Phyllostomus discolor	Brazil	2016
MH974777	Alphacoronavirus	Molossus rufus	Brazil	2017
MH974778	Alphacoronavirus	Molossus rufus	Brazil	2015
MT671955	Alphacoronavirus	Tadarida brasiliensis	Brazil	2014
MW465544	Alphacoronavirus	Desmodus rotundus	Brazil	2019
JQ731800	Alphacoronavirus	Molossus currentium	Brazil	2009
JQ731798	Alphacoronavirus	Carollia perspicillata	Brazil	2009
KU552073	Alphacoronavirus	Cynomops planirostris	Brazil	2013
KT717391	Alphacoronavirus	Myotis riparius	Brazil	2013
KT717390	Alphacoronavirus	Myotis nigricans	Brazil	2013
KX285054	Alphacoronavirus	Lichonycteris obscura	Brazil	2009
KX285055	Alphacoronavirus	Anoura caudifer	Brazil	2009
KX285224	Alphacoronavirus	Artibeus planirostris	Brazil	2011
MH974772	Alphacoronavirus	Phyllostomus discolor	Brazil	2015
MT671957	Alphacoronavirus	Tadarida brasiliensis	Brazil	2014
MW249018	Alphacoronavirus	Desmodus rotundus	Peru	2016
MW465545	Alphacoronavirus	Desmodus rotundus	Brazil	2019

KU552074	Alphacoronavirus	Glossophaga soricina	Brazil	2013
KU552076	Alphacoronavirus	Cynomops abrasus	Brazil	2013
KT717385	Alphacoronavirus	Carollia perspicillata	Brazil	2012
KX094982	Alphacoronavirus	Molossus molossus	Brazil	2011
KX285048	Alphacoronavirus	Carollia perspicillata	Bolivia	2011
KX285058	Alphacoronavirus	Glossophaga soricina	Brazil	2009
KX285060	Alphacoronavirus	Sturnira lilium	Brazil	2010
KX285063	Alphacoronavirus	Glossophaginae sp.	Brazil	2010
MH974769	Alphacoronavirus	Molossus rufus	Brazil	2015
MH974770	Alphacoronavirus	Molossus rufus	Brazil	2015
MN872698	Alphacoronavirus	Carollia perspicillata	Brazil	2012
JQ731799	Alphacoronavirus	Molossus rufus	Brazil	2009
KU552072	Alphacoronavirus	Desmodus rotundus	Brazil	2013
KT717380	Alphacoronavirus	Artibeus lituratus	Brazil	2010
KT717388	Alphacoronavirus	Molossus rufus	Brazil	2013
KT717382	Alphacoronavirus	Artibeus lituratus	Brazil	2012
KT717394	Alphacoronavirus	Sturnira lilium	Brazil	2012
KT717387	Alphacoronavirus	Glossophaga soricina	Brazil	2014
KX285057	Alphacoronavirus	Carollia perspicillata	Brazil	2009
KX285059	Alphacoronavirus	Sturnira lilium	Brazil	2010
MH974774	Alphacoronavirus	Phyllostomus discolor	Brazil	2016
MT671954	Alphacoronavirus	Tadarida brasiliensis	Brazil	2014
MN872694	Alphacoronavirus	Artibeus planirostris	Brazil	2012
JQ731794	Alphacoronavirus	Carollia perspicillata	Brazil	2009
KC886321	Alphacoronavirus	Molossus rufus	Brazil	2010
KU552079	Alphacoronavirus	Cynomops planirostris	Brazil	2014
KT717383	Alphacoronavirus	Carollia perspicillata	Brazil	2011
KX285053	Alphacoronavirus	Anoura caudifer	Brazil	2009
MH974766	Alphacoronavirus	Eptesicus sp.	Brazil	2015
MT671956	Alphacoronavirus	Tadarida brasiliensis	Brazil	2005
MN872693	Alphacoronavirus	Artibeus planirostris	Brazil	2012
MN872695	Alphacoronavirus	Artibeus lituratus	Brazil	2012
KM514667	Alphacoronavirus	Tadarida brasiliensis	Brazil	2011
OM265165	Alphacoronavirus	Artibeus lituratus	Brazil	2021
OM265166	Alphacoronavirus	Artibeus lituratus	Brazil	2021
OM265167	Alphacoronavirus	Artibeus lituratus	Brazil	2021
OM265168	Alphacoronavirus	Artibeus lituratus	Brazil	2021
OM265169	Alphacoronavirus	Artibeus lituratus	Brazil	2021
OM265170	Alphacoronavirus	Artibeus lituratus	Brazil	2021
OM265171	Alphacoronavirus	Carollia perspicillata	Brazil	2021
OM265172	Alphacoronavirus	Carollia perspicillata	Brazil	2021
OM265173	Alphacoronavirus	Carollia perspicillata	Brazil	2021
OM265174	Alphacoronavirus	Carollia perspicillata	Brazil	2021
OM265175	Alphacoronavirus	Carollia perspicillata	Brazil	2021
OM265176	Alphacoronavirus	Carollia perspicillata	Brazil	2021
OM265177	Alphacoronavirus	Carollia perspicillata	Brazil	2021
OM265178	Alphacoronavirus	Carollia perspicillata	Brazil	2021
OM265179	Alphacoronavirus	Carollia perspicillata	Brazil	2021
OM265180	Alphacoronavirus	Artibeus cinereus	Brazil	2021
OM265181	Alphacoronavirus	Diphylla ecaudata	Brazil	2021
OM265182	Alphacoronavirus	Sturnira lilium	Brazil	2021

OM265183	Alphacoronavirus	Phyllostomus hastatus	Brazil	2021
OM265184	Alphacoronavirus	Phyllostomus discolor	Brazil	2021
OM265185	Alphacoronavirus	Phyllostomus discolor	Brazil	2021
OM265186	Alphacoronavirus	Phyllostomus discolor	Brazil	2021
OM265187	Alphacoronavirus	Phyllostomus discolor	Brazil	2021
OM265188	Alphacoronavirus	Phyllostomus discolor	Brazil	2021
OM265189	Alphacoronavirus	Phyllostomus discolor	Brazil	2021
OM265190	Alphacoronavirus	Phyllostomus discolor	Brazil	2021
OM265191	Alphacoronavirus	Phyllostomus discolor	Brazil	2021
OM265192	Alphacoronavirus	Phyllostomus discolor	Brazil	2021
OM265193	Alphacoronavirus	Phyllostomus discolor	Brazil	2021
OM265194	Alphacoronavirus	Phyllostomus discolor	Brazil	2021
OM265195	Alphacoronavirus	Phyllostomus discolor	Brazil	2021
OM265196	Alphacoronavirus	Phyllostomus discolor	Brazil	2021
OM265197	Alphacoronavirus	Phyllostomus discolor	Brazil	2021
OM265198	Alphacoronavirus	Sturnira lilium	Brazil	2021
OM265199	Alphacoronavirus	Sturnira lilium	Brazil	2021
OM265200	Alphacoronavirus	Sturnira lilium	Brazil	2021
OM265201	Alphacoronavirus	Sturnira lilium	Brazil	2021
OM265202	Alphacoronavirus	Sturnira lilium	Brazil	2021
OM265204	Alphacoronavirus	Sturnira lilium	Brazil	2021
OM265203	Alphacoronavirus	Sturnira lilium	Brazil	2021
OM265205	Alphacoronavirus	Sturnira lilium	Brazil	2021
OM265206	Alphacoronavirus	Sturnira lilium	Brazil	2020
OM265207	Alphacoronavirus	Sturnira lilium	Brazil	2020
OM265208	Alphacoronavirus	Sturnira lilium	Brazil	2020

Clade A

	PD	SR
Molossus	247.71	18
Eptesicus	221.17	9
Glossophaga	85.02	1
Tadarida	91.77	3

**MPD**	ntaxa	mpd.obs	mpd.rand.mean	mpd.rand.sd	mpd.obs.rank	mpd.obs.z	mpd.obs.p	runs
Molossus	18	46.580	105.300	9.282	1.000	-6.326	0.001	1000
Eptesicus	9	82.602	105.239	17.524	110.000	-1.292	0.110	1000
Glossophaga	1	NA	NaN	NA	NA	NA	NA	1000
Tadarida	3	1.529	104.518	44.306	2.000	-2.324	0.002	1000

**MNTD**	ntaxa	mntd.obs	mntd.rand.mean	mntd.rand.sd	mntd.obs.rank	mntd.obs.z	mntd.obs.p	runs
Molossus	18	9.452	15.193	5.204	139.000	-1.103	0.139	1000
Eptesicus	9	12.751	26.989	11.700	142.000	-1.217	0.142	1000
Glossophaga	1	NA	NaN	NA	NA	NA	NA	1000
Tadarida	3	1.196	72.400	36.361	4.000	-1.958	0.004	1000

Phylogenetic Beta Diversity

	Molossus	*Eptesicus*	*Glossophaga*
Eptesicus	140.10		
Glossophaga	30.62	134.06	
Tadarida	175.10	128.94	175.38

Clade B

	PD	SR
Tadarida	101.03	20
Myotis	307.02	26
Perimyotis	59.19	1
Cynomops	74.91	6
Molossus	69.50	3

**MPD**	ntaxa	mpd.obs	mpd.rand.mean	mpd.rand.sd	mpd.obs.rank	mpd.obs.z	mpd.obs.p	runs
Tadarida	20	15.198	80.093	5.151	1.000	-12.597	0.001	1000
Myotis	26	44.253	80.012	3.853	1.000	-9.281	0.001	1000
Perimyotis	1	NA	NaN	NA	NA	NA	NA	1000
Cynomops	6	6.957	79.497	15.946	2.000	-4.549	0.002	1000
Molossus	3	0.366	77.872	30.903	1.000	-2.508	0.001	1000

**MNTD**	ntaxa	mntd.obs	mntd.rand.mean	mntd.rand.sd	mntd.obs.rank	mntd.obs.z	mntd.obs.p	runs
Tadarida	20	1.611	10.652	4.697	1.000	-1.925	0.001	1000
Myotis	26	11.729	8.826	3.276	799.000	0.886	0.798	1000
Perimyotis	1	NA	NaN	NA	NA	NA	NA	1000
Cynomops	6	3.800	26.367	16.176	54.000	-1.395	0.054	1000
Molossus	3	0.265	54.643	31.708	1.000	-1.715	0.001	1000

Phylogenetic Beta Diversity

	Tadarida	*Myotis*	*Perimyotis*	*Cynomops*
Myotis	113.95			
Perimyotis	118.23	61.68		
Cynomops	100.96	113.91	113.77	
Molossus	12.59	115.68	120.43	103.16

Supplementary Table S3. Phylogenetic diversity results. Metrics of CoV phylogenetic diversity within each bat genus for clades A and B. Phylogenetic diversity (PD); species richness (SR); standardized effect size of mean phylogenetic distance (SES MPD); standardized effect size of mean nearest taxon distance (SES MNTD); and phylogenetic beta diversity (among bat genera) are shown. The SES values were calculated by randomly reshuffling tip labels 1000 times along the entire phylogeny.
